# Analysis of related factors influencing the sleep quality in patients with rotator cuff tear after arthroscopic surgery

**DOI:** 10.1097/MD.0000000000039841

**Published:** 2024-09-27

**Authors:** Qian Zhang, Yanjun Li, Yongwang Li, Chunguang Wang, Yichao Yao, Qiuping Li

**Affiliations:** a Department of Orthopedics Surgery, The Baoding No.1 Central Hospital, Hebei, P. R. China; b Department of Anesthesiology, The Baoding No.1 Central Hospital, Hebei, P. R. China; c Department of Operating Room, The Baoding No.1 Central Hospital, Hebei, P. R. China; d Department of Radiology, The Affiliated Hospital of Hebei University, Hebei, P. R. China.

**Keywords:** disability evaluation, psychologic status, rotator cuff, shoulder, sleep disturbance

## Abstract

Recent studies have reported a prevalence of sleep disturbance in patients undergoing arthroscopic rotator cuff repair. The purpose of our study was to explore patient-reported factors correlated with sleep disturbance in patients with arthroscopic rotator cuff repair. We retrospectively evaluated 133 patients who underwent arthroscopic rotator cuff repair for 6 months. We obtained the Pittsburgh Sleep Quality Index (PSQI) scores, the visual analog scale (VAS) pain score, the University of California-Los Angeles Shoulder Rating Scale score (UCLA score), the Hospital Anxiety and Depression Scale (HADS), and patient demographics. According to the PSQI scores, participants were divided into a sleep disorder group (A group, PSQI ≥5) and a normal sleep group (B group, PSQI <5). Statistical analyses included Student *t* test, Mann–Whitney *U* test, chi-square test, and binary logistic regression analysis to determine which patient-reported factors were associated with sleep disturbance. The mean VAS, UCLA score, UCLA Flexion, HADS-Anxiety (HADS-A), and HADS-Depression (HADS-D) scores in group A were 3.54, 26.36, 3.25, 5.43, and 5.93, respectively; in group B, the mean scores were 1.49, 30.72, 4.50, 2.11, and 1.79, respectively. There were statistically significant differences in the VAS, UCLA, UCLA Flexion, HADS-A, HADS-D scores between the 2 groups (*P* < .05). In the categories of sex, age, body mass index, and tear size, there was no statistical significant difference between the 2 groups. (*P* > .05). HADS-D and UCLA Flexion were independent factors affecting sleep disturbance after arthroscopic rotator cuff repair at 6 months (*P* < .05). Our study demonstrated that patients with sleep disturbances after arthroscopic shoulder surgery had a close relationship with the HADS-D, UCLA Flexion scores and had more pain, more dysfunction, and more pronounced psychological abnormalities. Therefore, more emphasis on psychotherapy and rehabilitation is required for patients with sleep disturbance.

## 1. Introduction

Sleep disturbance is a common symptom in patients with rotator cuff tears. Its prevalence is high, ranging from 70.2% to 89%.^[1]^ Arthroscopic rotator cuff repair as an effective treatment for rotator cuff can significantly improve sleep quality; however, the incidence of poor sleep quality still ranges between 38% and 58%.^[2]^ Sleep has been considered beneficial as a dynamic form of energy conversation and reorganization of neuronal activity.^[3]^ A previous study showed that sleep disturbance negatively influences the quality of life.^[4]^ As such, sleep quality has received more attention for increasing the quality of life of patients undergoing rotator cuff repair.

The Pittsburgh Sleep Quality Index (PSQI) scores have been validated as an assessment tool for sleep quality to quantify abnormal sleep, and a PSQI score (range from 0 to 21) ≥5 is indicative of poor sleep quality.^[5]^ Several recent studies have reported that the mean PSQI score decreases after arthroscopic procedure.^[6–8]^ Nevertheless, sleep disturbance and nocturnal pain are still the main complications that reduce the quality of life of patients undergoing rotator cuff repair. Several studies have been performed on the relationship between the clinical outcomes of the visual analog scale (VAS) and PSQI.^[6,8]^ To date, there has been no consensus on the factors associated with sleep disorders in patients after arthroscopic surgery.

The aims of this study were to explore the ways in which patients with sleep disorders after rotator cuff surgery differ from patients with normal sleep and to estimate which demographic variables and patient self-rated outcome factors were associated with sleep disorders in patients after rotator cuff repairs.

## 2. Materials and methods

**Figure 1.**
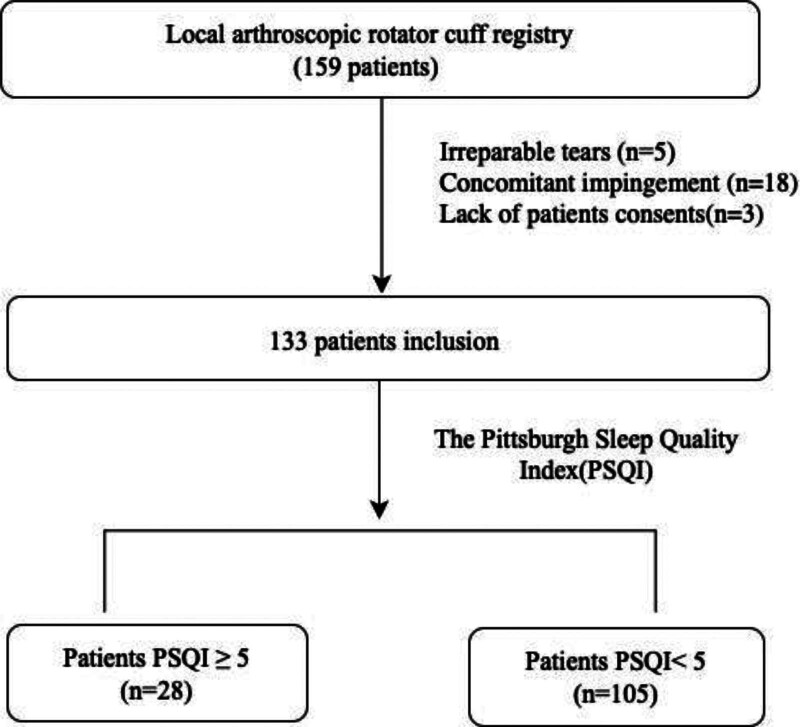
Patients selection flowchart.

We recruited 133 individuals who had undergone arthroscopic rotator cuff repair at our facility between September 2015 and June 2021 after obtaining approval from the institutional ethics review committee (The Baoding No.1 Central Hospital. ID:[2022]012). All procedures were performed by the same surgeon. Postoperative rehabilitation protocols were described as follows: 0 to 4 weeks shoulder abduction pillows sling, passive forward flexion, and pendulum exercise twice a day for 5 minutes, active range of motion of elbow, twist, hand; 4 to 8 weeks shoulder abduction pillows sling discontinued (6 weeks), passive forward flexion and pendulum exercise twice a day for 5 minutes, active range of motion of elbow, twist, hand. Eight to 12 weeks active-assisted range of motion to tolerance; after 12 weeks begin strengthen. The inclusion criteria were: age >18 years, arthroscopic rotator cuff repair, ruptures involving only the supraspinatus and/or infraspinatus, fatty degeneration level of 0 or 1 based on modified Goutallier classification. The exclusion criteria were history of other surgeries on the ipsilateral or contralateral shoulder; inability to complete the follow-up schedule and questionnaires; any history of psychotic or mood disorders that had been recorded; other diseases of the shoulder joint, such as osteoarthritis and shoulder impingement syndrome; concurrent pathology in labrum that requires surgery; concomitant pathology in the acromioclavicular joint that requires distal clavicle resection. The patients completed the follow-up questionnaires at postoperative 6 months. The PSQI scores, the VAS pain score, the University of California-Los Angeles Shoulder Rating Scale score (UCLA score), the Hospital Anxiety and Depression Scale (HADS) were all included in the questionnaires. Demographic and medical data were obtained by reviewing medical records, including age, sex, body mass index, comorbidities (hypertension, diabetes), acromiohumeral distance, and rotator cuff tear size. Based on their sleep conditions 6 months after surgery, all 133 patients were divided into 2 groups: a sleep disturbance group with a PSQI score ≥5 (A group, n = 28) and a normal sleep group with a PSQI score <5 (B group, n = 105) (Fig. 1).

### 2.1. Assessment and instruments

#### 2.1.1. PSQI score

The PSQI had been widely used as a reliable valid and standardized instrument for measuring subjective sleep quality in many orthopedic subspecialties.^[5,9,10]^ The global PSQI score ranges between 0 and 21, with 7 components. Based on previous studies, a PSQI score ≥5 is a valid indicator of poor sleep, with a sensitivity of 89.6% and a specificity of 86.5%.^[5]^ Higher PSQI scores indicated a higher magnitude of sleep disturbance.

#### 2.1.2. VAS pain score

VAS pain score is a subjective self-rating tool used to assess pain. The VAS score ranged from 0 (no pain) to 10 (unaffordable). The points rated by the participants indicated the degree of pain they experienced.

#### 2.1.3. UCLA Shoulder Rating Scale score

The UCLA shoulder scale is one of the most commonly used tools for assessing shoulder function and outcomes.^[11,12]^ It was composed of 5 components: pain, function, active forward flexion (UCLA Flexion), strength of forward flexion, and satisfaction of the patient.

#### 2.1.4. Hospital Anxiety and Depression Scale

The HADS is a self-report instrument used to evaluate depression and anxiety in clinical study.^[13]^ It included two 7-item subscales rated on a 4-point response scale: the Hospital Anxiety and Depression Scale anxiety (HADS-A) and the Hospital Anxiety and Depression Scale depression (HADS-D). Each scale score ranges from 0 to 21. The HADS is a reliable instrument to assess depressive and anxiety disorders that has been validated in orthopedic patients.^[14,15]^

### 2.2. Statistical analysis

To examine the patients’ baseline characteristics, frequency and descriptive statistics were analyzed. For continuous variables that conformed to a normal distribution, Student *t* tests were used to evaluate the differences between the sleep disorders group (A group) and the normal sleep group (B group), while those not conforming were assessed using the Mann–Whitney *U* test. For categorical variables, the chi-square test was used to evaluate differences. We then used binary logistic regression analysis to assess dichotomous independent variables. Statistical analysis was performed using SPSS software (version 23.0; SPSS Inc., Chicago, IL).

## 3. Results

In terms of sex, age, body mass index, and tear size, the sleep disturbance group did not differ significantly from the normal sleep group (*P* > .05).

The mean VAS, UCLA Score, UCLA Flexion, HADS-A, and HADS-D scores in group A were 3.54, 26.36, 3.25, 5.43, and 5.93, respectively, while those in group B were 1.49, 30.72, 4.50, 2.11, and 1.79, respectively. There was a statistically significant difference in the VAS, UCLA, UCLA Flexion, HADS-A, HADS-D scores between the 2 groups (*P* < .05). Minimal clinically important difference (MCID) is a vital tool in the analysis of clinical results. Studies by related scholars indicate the mean MCID value for the UCLA shoulder score was 3 to 3.5 points.^[16,17]^ In our study, between-group differences never exceeded the MCID of UCLA. For the Patient Acceptable Symptom State, the literature indicates that the Patient Acceptable Symptom State was 7 on the HADS, 3.5 on the HADS-A, and 3.5 on the HADS-D.^[15]^ Based on this criterion, in our study, when comparing the postoperative HADS scores between the 2 groups, there was only a statistically significant difference in HADS-A, with no clinical difference, whereas HADS-D showed both statistically significant and clinically significant differences (Table 1).

**Table 1:** Results of a univariate analysis affecting sleep quality after shoulder arthroscopic rotator cuff repair.

Characteristic	Sleep disorders group	Normal sleep group	*t*/*z*/*x*²	*P*
	N = 28	N = 105		
Age, y	55.21 ± 7.62	56.53 ± 8.79	−0.82	.409
Gender			0.013	.908
Male	11 (39.3)	40 (38.1)		
Female	17 (60.7)	65 (61.9)		
BMI (kg/m²)	25.01 ± 2.13	24.71 ± 2.23	−0.63	.524
Tear size			−1.95	.110
Small	9 (32.1)	45 (42.9)		
Medium	13 (46.4)	52 (49.5)		
Large	6 (21.4)	8 (7.6)		
Hypertension	12 (42.8)	56 (53.3)	0.24	.619
Diabetes	8 (28.5)	26 (24.7)	0.17	.672
VAS	3.54 ± 1.13	1.49 ± 0.86	−7.22	.000*
UCLA Score	26.36 ± 3.77	30.72 ± 2.79	−5.71	.000*
UCLA Flexion	3.25 ± 0.64	4.50 ± 0.60	−6.90	.000*
AHD	7.43 ± 0.27	7.51 ± 0.29	−0.80	.438
HADS-A	5.43 ± 1.37	2.11 ± 1.41	−7.38	.000*
HADS-D	5.93 ± 1.12	1.79 ± 1.55	−7.77	.000*

Continuous data are shown as the mean ± standard deviation and categorical data as number (%); statistical significance was set at *P* < .05.

AHD = acromiohumeral distance, BMI = body mass index, HADS-A = Hospital Anxiety, and Depression Scale anxiety subscale, HADS-D = Hospital Anxiety and Depression Scale depression subscale, PSQI = Pittsburgh Sleep Quality Index, UCLA Flexion = the forward flexion score of The University of California-Los Angeles (UCLA) Shoulder Rating Scale score, UCLA score = The University of California-Los Angeles (UCLA) Shoulder Rating Scale score, VAS = visual analog scale.

After a binary logistic regression analysis of the variables with a statistically significant difference, the results showed that HADS-D and UCLA Flexion were independent factors affecting sleep disturbance after arthroscopic rotator cuff repair for six months (*P* < .05; Table 2).

**Table 2:** Results of binary logistic regression analysis affecting sleep quality after shoulder arthroscopic rotator cuff repair.

Variable	*B*	OR	95% CI	*P*
VAS	2.429	11.353	0.450–286.658	.140
UCLA Score	-0.245	0.782	0.430–1.425	.422
UCLA Flexion	-2.546	0.078	0.009–0.666	.020*
HADS-A	-0.105	0.900	0.306–2.647	.849
HADS-D	1.976	7.212	1.310–39.698	.023*

*B* = regression coefficient *b* value, statistically significant at *P* < .05.

CI = confidence interval, HADS-A = Hospital Anxiety, and Depression Scale anxiety subscale, HADS-D = Hospital Anxiety and Depression Scale depression subscale, OR = odds ratio, UCLA Flexion = the forward flexion score of The University of California-Los Angeles (UCLA) Shoulder Rating Scale score, UCLA score = The University of California-Los Angeles (UCLA) Shoulder Rating Scale score, VAS = visual analog scale.

## 4. Discussion

Previous studies have shown that 38% of patients still had sleep disturbances after arthroscopic rotator cuff repair, although sleep conditions were significantly improved.^[18]^ The purpose of our study was to explore the differences between patients with sleep disorders and those with normal sleep after rotator cuff surgery, and to evaluate which factors influence sleep disturbance in patients after arthroscopic rotator cuff repair. These results provide a partial reference for improving the sleep status of patients undergoing rotator cuff repair.

Our results demonstrated that patients with sleep disturbances after arthroscopic rotator cuff repair had higher VAS, HADS-A, and HADS-D scores, and lower UCLA, UCLA Flexion scores than those with normal sleep. This variability by sleep disorder or not revealed a possible close relationship between sleep disorders and pain, disability, depression, and anxiety. This relationship has also been reported in previous studies and is attributed to a model of adverse interaction between psychological state and chronic pain.^[19–21]^

A study by Cho et al in patients with a history of rotator cuff disease for more than 3 months showed results that coincide with our results that patients with higher PSQI scores had higher VAS pain scores and HADS-A and HADS-D scores, and lower self-reported function outcomes.^[6]^ In a follow-up study of 37 patients 2 years after rotator cuff repair, Horneff et al^[8]^ also found that PSQI scores, patient self-rated outcomes, and VAS pain scores continued to improve as postoperative time increased. Patients with sleep disorders have relatively high pain and functional scores.^[8]^ A recent prospective study showed similar results in that the postoperative PSQI correlated with the 36-Item Short Form Survey, American Shoulder and Elbow Surgeons Shoulder Score, and Constant-Murley Score.^[22]^ This study divided patients after rotator cuff repair into 2 groups based on their PSQI scores to investigate the differences between the 2 groups and evaluate the factors influencing sleep disorders.

Furthermore, many studies have not reached consensus. Bingöl and Biçici^[23]^ found that the postoperative PSQI scores were significantly lower in patients with small tears than in those with medium- and large-size tears. However, other researchers have found that patients with small tears have poorer sleep quality than those with more severe tear.^[24]^ In our study, no statistical difference in tear size of the rotator cuff was found between the 2 groups.

Although our study indicated that VAS, UCLA, UCLA Flexion, HADS-A, and HADS-D scores were statistically different between the 2 groups, only UCLA Flexion and HADS-D scores were significantly correlated with the PSQI after a binary logistic regression analysis. Mulligan et al^[25]^ conducted a clinical study involving 343 patients. They found that patients with adhesive capsulitis were particularly vulnerable to reduced sleep quality. This finding is consistent with our results. The UCLA Flexion scores, as an indicator of the magnitude of joint mobility, were not only statistically different between the sleep disturbance group and the normal sleep group but were also significantly associated with sleep disturbance. Cho et al found that sleep disturbance was closely related to depression and anxiety in patients with shoulder pain for 3 months or longer.^[6]^ A study evaluating the correlation between rotator cuff tear size and sleep disturbance demonstrated that the size of full-thickness rotator cuff tears did not correlate with sleep disturbance and had little to no correlation with pain levels.^[26]^ Similarly, other studies have shown that sleep quality was not significantly correlated with tear size.^[27]^ Our results are consistent with those of previous studies on patients with rotator cuff tendinopathy.

From a clinical perspective, our results show that in patients with sleep disorders after rotator cuff repair, a more specific rehabilitation program and more attention to psychology would help improve their sleep status.

Our study had several limitations. First, the results may not be generalizable to all patients from the same institution. Second, there was a response bias because our assessment of psychological status and sleep disturbance were subjective results obtained by a questionnaire and based on participants’ self-reports of their status.

## 5. Conclusions

Based on our data, patients with sleep disturbances after arthroscopic shoulder surgery had severe pain, more obvious dysfunction, and more pronounced psychological abnormalities. The close relationships between depression, UCLA Flexion scores, and sleep disturbance may indicate that more emphasis on psychotherapy and rehabilitation is required. In the future, it will be necessary to conduct a multicenter study with a larger sample size to validate generalizability.

## Author contributions

**Conceptualization:** Qian Zhang, Yanjun Li.

**Methodology:** Qian Zhang, Yichao Yao, Qiuping Li.

**Writing – original draft:** Qian Zhang, Yongwang Li.

**Investigation:** Yanjun Li, Yichao Yao, Qiuping Li.

**Formal analysis:** Chunguang Wang.
